# An Incremental Class-Learning Approach with Acoustic Novelty Detection for Acoustic Event Recognition

**DOI:** 10.3390/s21196622

**Published:** 2021-10-05

**Authors:** Barış Bayram, Gökhan İnce

**Affiliations:** 1 Computer Engineering Department, Faculty of Computer and Informatics Engineering, Istanbul Technical University, Istanbul 34469, Turkey; baris.bayram@itu.edu.tr; 2 Artificial Intelligence and Data Science Application and Research Center, Istanbul Technical University, Istanbul 34469, Turkey

**Keywords:** acoustic scene analysis, acoustic event recognition, acoustic novelty detection, audio signal augmentation, incremental class-learning

## Abstract

Acoustic scene analysis (ASA) relies on the dynamic sensing and understanding of stationary and non-stationary sounds from various events, background noises and human actions with objects. However, the spatio-temporal nature of the sound signals may not be stationary, and novel events may exist that eventually deteriorate the performance of the analysis. In this study, a self-learning-based ASA for acoustic event recognition (AER) is presented to detect and incrementally learn novel acoustic events by tackling catastrophic forgetting. The proposed ASA framework comprises six elements: (1) raw acoustic signal pre-processing, (2) low-level and deep audio feature extraction, (3) acoustic novelty detection (AND), (4) acoustic signal augmentations, (5) incremental class-learning (ICL) (of the audio features of the novel events) and (6) AER. The self-learning on different types of audio features extracted from the acoustic signals of various events occurs without human supervision. For the extraction of deep audio representations, in addition to visual geometry group (VGG) and residual neural network (ResNet), time-delay neural network (TDNN) and TDNN based long short-term memory (TDNN–LSTM) networks are pre-trained using a large-scale audio dataset, Google AudioSet. The performances of ICL with AND using Mel-spectrograms, and deep features with TDNNs, VGG, and ResNet from the Mel-spectrograms are validated on benchmark audio datasets such as ESC-10, ESC-50, UrbanSound8K (US8K), and an audio dataset collected by the authors in a real domestic environment.

## 1. Introduction

Due to recent breakthroughs in deep learning and advancements in artificial intelligence, deep neural networks (DNNs), powerful learning models inspired by biological neural networks, have been developed to deal with many problems in computer vision, signal processing, and natural language processing. One of the popular deep learning challenges is human and animal-like lifelong learning also known as incremental/continual learning; that is, learning without storing and retraining entire previous data due to resource limitations of space and computational complexity. However, traditional DNNs are generally prone to “catastrophic forgetting” [1,2], in which previously seen instances, classes or tasks may be forgotten. Therefore, for incremental class learning (ICL) in the last few years, the learning of new classes, or tasks, in a different domain using architectural, regularization, and rehearsal strategies, in combination or independently, has attracted considerable attention to satisfy the requirement of sequentially learning without forgetting and re-using previously learned data [3].

Most of ICL works have focused on computer vision tasks such as image classification [4], semantic segmentation [5], image classification in a number of isolated tasks [6]. Only a few [7,8] have focused on the incremental learning of new acoustic events for detection of the events. However, incremental learning without forgetting may also be useful for various tasks such as speech recognition, voice detection, acoustic scene analysis (ASA), acoustic event recognition (AER), acoustic anomaly detection (AAD), acoustic novelty detection (AND). The tasks of AER and AND in acoustic scenes have not received as much attention as speech recognition, but the significance of the scene analysis using audio signals has been demonstrated in a variety of applications for surveillance [9], elderly human monitoring [10], home automation [11], and robotics [12]. Environmental sounds differ from speech and musical sounds especially in temporal structure and spectrum frequency [13]. Also, these sounds have noise-like characteristics with a broad flat spectrum that adversely affects the recognition of environments using Mel-frequency cepstral coefficient (MFCC)-type features [14]. For learning the non-stationary and dynamic nature of acoustic signals of events in different environments, several works have focused on algorithms under the difficulties of environmental sounds. Audio features including low-level ones (e.g., zero-crossing rate and short-time energy and complex high-dimensional features (e.g., MFCCs [15], Mel-spectrograms [16], gammatone–spectrograms [17], and wavelet-based features [18]) have been used in the literature. Spectrograms from raw audio signals are used to represent the temporal and spectral structure of the signal, and representative DNNs, such as ResNet [19] and VGG [20], extract deep-audio representations from images such as Mel-spectrograms for AER and AAD.

In real-world tasks, a large amount of annotated data may not be available due to its nature and the expense of annotation. Also, in various acoustic tasks and environments, novel events or unknown noises may appear that exacerbate ASA performance. Therefore, it is necessary to detect novel acoustic events and learn the event incrementally to enhance the recognition capability for acoustic tasks. However, after detecting a novel acoustic event, only a few samples have been observed to be used in ICL and in the retraining of the semi-supervised novelty detection method. The scarcity of data is always an important challenge in AER tasks. Audio signal augmentation is applied by time-stretching to increase the amount of audio samples belonging to the detected novel acoustic event [21]. In this study, ICL with novelty detection based ASA for AER is presented and various algorithms are investigated using different audio features to achieve the ICL and AND tasks.

To extract deep audio representations from acoustic events, inn this work different deep networks were pre-trained using a large scale of audio datasets. Time-delay neural networks (TDNNs) were used to recognize speech, speech emotion, and speaker and to detect of voice activity. Moreover, TDNN-based approaches are sufficient for capturing the complex temporal characteristics of environmental sounds with transient, intermittent, and continuous temporal structures. Therefore, factorized TDNN (F-TDNN) [22] and TDNN with long-short term memory (LSTM) [23] were applied to MFCCs and accoustic event raw signals, respectively. In addition to the TDNNs, ResNet and VGG were pre-trained on AudioSet [24], including a large amount of audio data and then employed on different benchmark datasets to extract the deep audio representations. Moreover, for AND, the following state-of-the-art methods were applied in a semi-supervised manner: stacked autoencoder (AE) [25], variational AE (VAE) [26], k-nearest neighbour (kNN) [27], Gaussian mixture model (GMM) [28], one-class support vector machine (OCSVM) [29], and isolation forest (iForest) [30]. To achieve ICL, learning without forgetting (LwF) [31], an incremental classifier and representation learning (iCaRL) [32], and FearNet [33] were employed on these five types of audio features: Mel-spectrograms, and deep features from TDNN, TDNN–LSTM, ResNet, and VGG.

The contributions of this work can be listed as follows;

1. the use and investigation of ICL algorithms using acoustic signals in an AER task,
2. the pre-training of F-TDNN and TDNN-LSTM using MFCCs and raw acoustic signals, respectively,
3. the extraction of deep audio representations with the pre-trained F-TDNN and TDNN-LSTM,
4. the development of a semi-supervised AND method to detect new acoustic events for ICL,
5. the augmentation of audio signals to increase the number of features from the detected novel event for ICL and retraining of the AND algorithm,
6. the comparison of the deep features from the F-TDNN and TDNN-LSTM, and the state-of-the-art networks VGG-16 and ResNet-34 pre-trained using Mel-spectrograms from the same dataset,
7. the integration of ICL and AND in a single framework to achieve ICL without human supervision and
8. the collection of an audio dataset in a domestic environment.

To the best of our knowledge, the contributions regarding ICL with AND in acoustic tasks and pre-training of several networks for ICL and AND appear here for the first time in the literature. The proposed approach for ICL with AND was evaluated on the benchmark audio datasets ESC-10, ESC-50 [34], UrbanSound8K (US8K) [35], and the audio dataset (Domestic) collected by the authors using a microphone array located 1 m from the sound sources. The dataset was generated to achieve audio–visual and robotic tasks and investigate the microphone array in a domestic real environment.

The rest of the paper is organized as follows: Section 2 is a discussion of the related work in AER, DNNs to extract audio features, novelty detection in audio features, and ICL. The steps of the proposed ASA approach for ICL with AND are given in Section 3. The implementation details of the techniques and algorithms used for feature extraction, AND and ICL, and the experiments are provided in Section 4. The performances of stacked AE, VAE, kNN, GMM, iForest, and OCSVM for novelty detection, and LwF, iCaRL, and FearNet for ICL on five different types of audio features, Mel-spectrograms, and deep features from TDNN, TDNN-LSTM, VGG-16, and ResNet-34 are discussed in Section 5. Finally, the conclusion and future work are given in Section 5.

## 2. Background

In recent years, significant attention has been paid to the use of deep-learning-based approaches for ASA to deal with several audition tasks such as acoustic scene classification (ASC), acoustic event recognition (AER), acoustic novelty detection (AND), and acoustic anomaly detection (AAD) using different types of audio features. Furthermore, several deep-network-based studies have been proposed for incremental class-learning (ICL) in different domains. This section describes works on the use of different audio features for ASA, augmentation of audio data, detection of novel acoustic objects and events, and incremental learning of new classes.

### 2.1. Acoustic Scene Analysis

Various types of audio features have been employed to achieve ASA tasks (ASC, AAD, AND, and AER), which are composed of different types of time-frequency domain, cepstral and low-level audio features (Gammatone cepstral coefficients, MFCCs and variants [15], log-frequency filter bank coefficients [36], Mel-spectrograms [16], and a combination of the MFCCs and Mel-filter bank features [37]) have been used for ASC.

Many works have shown the substantially increased performances of deep neural networks (DNNs) using large datasets for AER. For the extraction of high-level feature representations, a deep belief network using spectrograms was proposed [38]. For transfer learning, a DNN was trained on a large dataset of a particular task, and then the network was applied to extract audio feature representations for a different dataset [21]. In the other DNN based work for AER, a CNN based approach was utilized on Mel-spectrograms [39]. As the input of the ResNet network, three-channel (RGB) images were used, so the spectrograms were converted to RGB images. Another study in which MFCCs were used as the input of a DNN was developed to construct x-vector embeddings for speaker verification tasks [40]. In addition, in several works, the TDNNs were exploited to recognize speech [41], emotion [42], speaker, or voice activity. However, the performances of TDNNs have not been investigated using sounds in different acoustic environments. To enhance the performance of sound event detection, other DNN-based acoustic techniques such as noise reduction [43], and dereverberation and beamforming [44] have been investigated. In our study for AER and ICL, the effectiveness of transfer learning was demonstrated using TDNNs pre-trained on the sounds of AudioSet to extract the embedding of acoustic events.

For the augmentation of audio data, a few works have been presented, in which augmentation was performed on raw audio signals, spectrograms, and low-level audio features. The spectrograms extracted from the sounds in the ESC-50 dataset were augmented and used to generate a CNN-based ensemble method [45]. Its performance was compared to many state-of-the-art CNN networks used only for feature extraction including variants of ResNet and VGG, AlexNet, GoogleNet, and Inception. Pandeya et al. used a domestic cat sound dataset to augment the raw audio signals in the dataset by randomly time-stretching, pitch-shifting, inserting noises of different ranges and dynamic range compression [46]. The augmentation was also applied to the raw audio signals and the spectrograms extracted from the signals in an audio dataset of natural animal sounds to improve the classification of animal sounds [47].

### 2.2. Novelty Detection on Acoustic Signals

Another challenging problem for ASA is to detect novel scenes in which unknown acoustic events occur. For AND, unsupervised deep networks and traditional one-class anomaly detection methods have been developed by the training data of known classes [26,48,49]. Nguyen et al. proposed a semi-supervised method based on a convolutional VAE that used to detect anomalous sounds [50]. The deep feature representations extracted by ResNet and VGG from audio images such as Mel-spectrograms have been exploited for abnormal sound detection [51]. Hoang et al. proposed four DNNs for AAD where an audio feature vector was constructed from MFCCs, Mel-Spectrogram, Spectral Contrast, Short-Time Fourier Transform, and Chroma features used for different autoencoders to apply anomalous sound detection [52]. We employ MFCCs widely used in acoustic tasks corresponding to transformed log filter-bank energies by a discrete cosine transform [53]. In addition, an algorithm for few-shot learning was developed to detect rare sounds in background noises [54]. Furthermore, sequential AEs were used for AAD in industrial acoustic environments [55]. Even fewer studies have been conducted in the area of few-shot learning to detect new acoustic events. Shi et al. [56] presented a few-shot method based on meta-learning for acoustic event detection.

### 2.3. Incremental Class-Learning

Various ICL algorithms have been developed using architectural, regularization, and rehearsal strategies, in combination or independently, to learn classes incrementally while avoiding the problem of catastrophic forgetting. The major problem of incremental learning was investigated in several studies in which different types of deep networks were proposed. After learning a new class, the parameters of the new model should not deviate too far from the configurations of the previous model. One of the oldest ICL algorithms based on CNN is learning without forgetting (LwF) [31], which focuses on transferring knowledge to overcome forgetting. Another ICL method called “incremental classifier and representation learning” (iCaRL), based on rehearsal and regularization approaches, was proposed in [32] using an external memory of a fixed size for previous exemplars. iCaRL used a nearest-exemplar algorithm for classification and relied on preventing substantial changes using the memory. However, the iCaRL algorithm, which has been shown to provide the best ICL performance in many studies, requires the storage of knowledge from the learned classes. A generative approach known as FearNet [33], based on a brain-inspired, dual-memory system achieves incremental learning of new classes by storing detailed statistics about known classes instead of the previous knowledge. For its incremental learning process, three short- and long-term memory networks and a decision network are exploited to choose the activated network. A few recent works have focused on incremental learning using CNN for AER. In [7,8], the performances of incremental learning were evaluated using Mel-spectrograms from one-second audio files. However, the detection of novel classes in the solutions to incremental learning, has not been addressed in the ICL studies. Furthermore, the works did not investigate widely used ICL algorithms and analyses of different audio features. To the best of our knowledge, ICL with AND has not yet been explored in the acoustic domain.

The lifelong learning problem has been widely studied using various types of machine learning. Some studies focused on combining it with novelty detection and few-shot learning. In one of these works [57], an incremental approach with novelty detection based on a Parzen window kernel density estimator was proposed. This method was applied to data streams regarding gestures to cope with the problems of real-time data streams, such as concept drift and the existence of novel classes. Also, class-based incremental learning [58] has been proposed, in which new classes are incrementally added to the model without forgetting the known classes. Ren et al. proposed incremental few-shot learning based on a meta-learning method (an Attention Attractor Network) to achieve few-shot learning incrementally without retraining the data [59].

## 3. Proposed Approach

**Figure 1:**
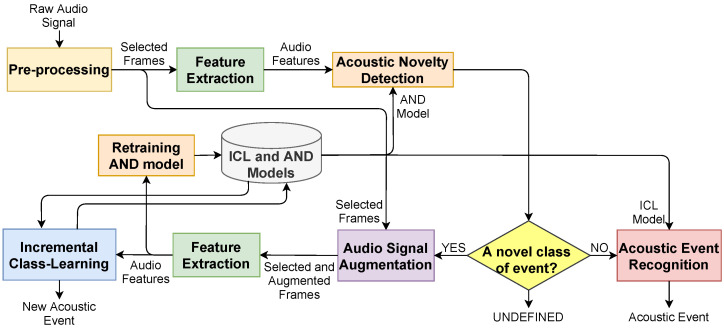
The block diagram of the proposed approach for incremental class-learning with novelty detection.

In this section, we introduce the proposed approach composed of six steps; (i) pre-processing, (ii) extraction of audio features, (iii) AND, (iv) augmentation of audio signals, (v) ICL, and (vi) AER (Figure 1). The main goals in the AND, ICL, and AER steps are to learn the function of

1. a novel event detector retrained in a semi-supervised manner and
2. an acoustic event recognizer that can learn incrementally from new events detected in the AND step.

### 3.1. Problem Definition

To analyze and model an audio signal, windowing may provide more accurate and robust acoustic measures for segmentation of distinctive characteristics from the audio sample. The most popular windowing technique in this work is the sliding/moving window with a fixed length through the signal. Let $S_{i}$ be the raw signal of an acoustic event recording, *i*, segmented by the sliding window with a constant temporal length (400 ms) (also called a frame). Then, $W_{i}^{k}\in R^{T}$, where *k* is the index of the window, and *T* denotes the window size, which is the time dimension. The length is selected and several tests are conducted while preserving feature stability and event information. Each window, $W_{i}^{k}$ is 50% overlapped by $W_{i}^{k-1}$ where $k=2,3,\cdots ,N_{i}$, and $N_{i}$ is the number of windows from the signal of the event recording, $S_{i}$. In the pre-processing step, after the windowing of the raw signal, window-based segmentation is applied to detect the ratio of silence and obtain the presence of sound events within all the 1-D windows, $W_{i}^{k}$. The sum of the samples for each window is compared with the pre-defined silence threshold close to 0 indicating the absence of any sound activity;

(1) $$\upsilon_{i}^{k}=\sum_{t=1}^{T}W_{i}^{k}\left(t\right),$$

where *t* denotes the time index in the samples of the *k*th window. Each window of the samples, the sum of which, $\upsilon_{i}^{k}$, is bigger than the silence threshold is selected for feature extraction.

### 3.2. Pre-Processing

In the pre-processing step, in addition to the windowing and window selection explained in Section 3.1, the optimum values of the parameters are selected: the overlapping factor for windowing, the number of coefficients for the extraction of MFCCs, the window size in short-time Fourier transform and the hop size for the extraction of Mel-spectrograms. In addirtion, the optimal setting of the parameters for the algorithms is determined.

### 3.3. Feature Extraction

In the feature extraction step, five feature types—deep audio representations of four pre-trained networks and a Mel-spectrogram—are extracted using the audio feature, $X_{i}^{k}\in \left\{I_{i}^{k},C_{i}^{k},W_{i}^{k}\right\}$. Each window is used to extract MFCCs, $C_{i}^{k}$ (a fixed number of d-dimensional feature vectors) and a Mel-spectrogram, $I_{i}^{k}$. The audio feature, $X_{i}^{k}\in \left\{I_{i}^{k},C_{i}^{k},W_{i}^{k}\right\}$ is used to extract the deep audio features with 128 dimensions; $D_{i}^{k}$ TDNN, TDNN-LSTM, VGG; and ResNet networks pre-trained with AudioSet, a large-scale dataset.

The F-TDNN, a deeper network than TDNN has four more channels, and the weight matrix of each TDNN layer is factorized by multiplying two smaller matrices to reduce the number of parameters in the layers [60,61]. Instead of singular value decomposition in a traditional DNN, the factorized architecture is employed for the reduction and the fine-tuning of the parameters after the reduction. In the TDNN–LSTM architecture, two TDNN layers are replaced by LSTM layers. MFCCs, $C_{i}^{k}$ of acoustic events, and the windows of the signal, $W_{i}^{k}$ are used, respectively, as the input of the F-TDNN and TDNN–LSTM networks instead of spectrograms like VGG-16 and ResNet-34. The Mel-spectrogram is also directly used in AND, AER, and ICL tasks. Finally, we conducted AND and ICL experiments to compute the contribution of these five feature types to the performance of AND and ICL. The most appropriate feature types were selected for use in the ICL with AND experiment.

### 3.4. Acoustic Novelty Detection

The AND step of the proposed method aims to detect novel acoustic events. The module has three possible outcomes ( “known”, “unknown” or “undefined”) depending on the novelty scores provided by the method and a two-level threshold strategy (Figure 1). An audio sample of an event is detected as “unknown” if it significantly deviates from a pre-defined decision threshold for novelty according to its novelty scores. A function of the novelty detector ($f_{and}:X_{i}^{k:N_{i}^{\prime }}$→$G_{i}$, where $N_{i}^{\prime }$ denotes the number of selected windows) is to learn to compute a novelty score, $G_{i}$ of the raw signal, $S_{i}$ for AND. The average of the novelty detection outputs of the selected features, $X_{i}^{k:N_{i}^{\prime }}$ is calculated to obtain a scalar novelty score for each acoustic event recording, *i*. The detector is defined on the audio feature set of the known events, and the AND model of the detector is retrained by including the features of the recently detected novel event.

In case a score greater than the threshold for AND was obtained, it was compared with another threshold for AER to improve the precision of the ICL and AND, and to prevent the propagation of the error. If the score was less than or equal to the threshold for AER, the audio sample was detected as “undefined”. Otherwise, the sample was presumed to be a “known” event and transmitted to the AER step for the prediction of its event class. The two-level thresholding strategy was adopted since false positives of AND might have negatively affected the performances of the ICL and AND algorithms.

A recently detected new acoustic class will inevitably have limited knowledge, which means sparsity may deteriorate the ICL and AND performances and cause over-fitting of ICL and AND models, or the forgetting of the old classes. To tackle the data sparsity problem, augmentation was directly applied to the raw audio signal of the new class, by time-stretching with randomly selected factors to increase the number of class samples. The stretching method changed the duration of the signal while preserving its spectral characteristic. The AND model was retrained by including new features of the selected windows from the actual and augmented signals in the previous training feature set. In the ICL step, only the features are incrementally learned.

ITo select the most appropriate AND method, six state-of-the-art one-class learning methods (stacked autoencoder (AE), variational AE (VAE), k-nearest neighbour (kNN), Gaussian mixture model (GMM), one-class support vector machine (OCSVM), and isolation forest (iForest)) were implemented. An AE is an unsupervised DNN comprised of an encoder and a decoder, which learns the input data to reconstruct robustly, so the AE is trained using the features of the known classes to detect a sample as a novel class that is not reconstructed well. In this work, a stacked AE, consisting of multiple AEs in a stacked form, and a VAE (a deep generative network combined with a statistics learning method to obtain a Gaussian mixture-like model) were used for AND. The stacked AE and VAE reconstructed the selected features, and the novelty score was the reconstruction error between the input features and the output of the AE networks. Moreover, these thresholds were automatically computed using the errors for AEs and novelty scores for the rest, and they identified for each algorithm after conducting several experiments for each dataset.

### 3.5. Incremental Class-Learning

Incremental learning is the only solution for learning from streaming or ephemeral data in which the entire dataset is required to be stored in memory to learn from scratch when new information exists [6]. For incremental learning the traditional neural networks are prone to one of the most important bottlenecks: catastrophic forgetting. The forgetting problem is related to the plasticity–stability dilemma [62] which occurs if a deep network is too plastic: the previously learned information is forgotten, and if the network is too stable, new information is not adequately learned. Therefore, to overcome the problem, various methods have been developed using the architectural, regularization and rehearsal strategies in combination or independently. Architectural strategies are aimed at learn new classes or information while maintaining previous knowledge. To avoid forgetting, the regularization strategy focuses on constraints on weight updates. Also, the rehearsal strategy is based on keeping a number of samples of the known classes instead of the entire data. In this study, audio features from selected windows of the original and augmented signals of new detected acoustic events are adapted to the ICL model. Unlike other ICLs, there is no human supervision for samples of a new class, so the algorithm is integrated with an AND method.

In the ICL step, an initial supervised setup for training includes a number of audio features, ${\left\{X_{i}^{k},y_{i}^{k}\right\}}_{e}$, extracted from a randomly selected recording of a randomly selected acoustic event, *e* in which *i* is the index of the recording, and *y* is the label of the event. For ICL, the audio samples of unknown events are sequentially and disjointedly learned while avoiding the forgetting problem. Therefore, a function of an incremental acoustic event recognizer, $f_{icl}:X_{i}^{k}\to y_{i}^{k}$ is learned.

We implemented a number of ICL methods (LwF, iCaRL, and FearNet) to investigate the performance of the incremental learning of novel classes with a special focus on the forgetting problem. In the LwF algorithm with an architectural strategy, the effect of forgetting was reduced by adding a term to the loss function of the network for the knowledge distillation to make the network output of new classes close to the original network output. iCaRL, which is based on a strategy of a combination of regularization and rehearsal, is also a incremental-class learner that is used to classify audio features by a nearest exemplar algorithm, and prevent catastrophic forgetting in the acoustic domain. The last ICL algorithm, FearNet [33], is based on a dual-memory system inspired from mammalian brains to learn new samples in short-term memory by a hippocampal network, and progressively consolidate them in long-term memory using pseudorehearsal [2] with a medial prefrontal cortex (mPFC) network. In addition, the basolateral amygdala, which is the third network, is exploited to decide whether to use the hippocampal or mPFC network for a sample.

## 4. Results and Discussion

### 4.1. Experimental Setup

For the implementations, Scikit-learn, a python package for three novelty detection techniques (GMM [28], OCSVM [29], and iForest [30]) and evaluation of the performances, and Keras for AEs and PyTorch for the ICL networks were used. Also, Librosa [63], another python package for audio analysis and signal processing, was used for basic operations for the extraction of MFCC and Mel-spectrograms. The domestic dataset was collected by a Kinect microphone array. Specifically, the pre-training of F-TDNN, TDNN-LSTM, VGG and ResNet networks was run on a machine with Intel® Core™ i7-8700K CPU and Nvidia GeForce, GTX 1080Ti GPU.

### 4.2. Experimental Procedure

In the experiments, three benchmark audio datasets (ESC-10, ESC-50 [34], UrbanSound8K (US8K) [35]) and our domestic audio dataset were used to evaluate the performances of (1) algorithms with the aforementioned feature types for novelty detection, (2) algorithms with the feature types for ICL, and (3) selected ICL model and feature types for ICL with AND. ESC-10 is a subset of the ESC-50 dataset which consists of 5 different sound categories: animal, non-speech human, urban or outdoor, indoor, and natural. Each sound clip in this dataset was 5 s long with a sampling frequency of 44,100 Hz. ESC-10 comprised 10 classes from these categories (dog barking, rain, sea waves, baby crying, clock ticking, person sneezing, helicopter, chainsaw, rooster, and fire crackling). The other benchmark dataset, US8K included short audio clips of up to 4 s from indoor and outdoor environmental sounds. Finally, our domestic audio dataset comprised 436 short clips of 10 domestic events (opening and closing doors, footsteps, taking a shower, kettle whistling, vacuum cleaner, cooking, dishwasher, toilet flushing, washing machine) with a duration between 1 and 12 s with a sampling frequency of 44,100 Hz which was non-overlapping.

The MFCC with 20-dimension and Mel-spectrogram features was extracted for each selected window from the acoustic signal processed at sampling rate of 44,100 Hz, where the parameters were set as follows: window size of 400 ms, step size of 200 ms (overlap factor of 50%), and FFT size of 512.

In the experiments of AND and ICL, the performances of the algorithms with the audio feature types were analyzed to estimate the most informative feature representations. Therefore, the pre-trained TDNN, TDNN-LSTM, VGG-16, and ResNet-34 models were initialized for transfer learning using a subset of the AudioSet including 5800 h of video clips with an ontology of 527 types of sound events from YouTube. The subset consisted of 40 classes of the environmental, urban and domestic categories. For AND and ICL, the deep audio representations were extracted by VGG-16 and ResNet-34 from Mel-spectrograms, and by F-TDNN and TDNN-LSTM from the MFCCs and raw signal, respectively, of the sound samples in the ESC-10, ESC-50, US8K, and Domestic datasets.

The first experiment had several experimental setups with many scenarios in which a different number of known or unknown classes were used to compare the AND algorithms (stacked AE, VAE, kNN, GMM, iForest, and OCSVM) to find the most promising algorithms. In the scenarios for the ESC-10, US8K, and Domestic dataset classes, 1, 3, 5, and 7 were known, and the rest of the 10 were unknown, and one where only 1 class was unknown. For ESC-50, a different experimental setup was performed. It had scenarios in which 1, 5, 10, 20, 30, 40, and 45 event classes were known and the rest were unknown, and one where only 1 class was unknown (the rest of the 49 event classes were known). In the last experiment, the proposed approach for ICL with AND on the acoustic data is investigated in which the most suitable AND and ICL algorithms are applied to the best performing audio feature representations.

### 4.3. Evaluation Metrics

To measure the performances of the AND algorithms, the average area under the curve (AUC) and F1-scores were calculated for each feature type and algorithm in each dataset. The AUC metric was generated by plotting the true positive rate vs. the false positive rate, and the AUC was computed from the success plot. The F1-scores were computed as the harmonic mean of precision and sensitivity (recall). The average of the accuracies in a test set, randomly selected from each dataset and the average F1-score, was used to assess the performances of the algorithms with the feature types for ICL and ICL with AND.

### 4.4. Results of Novelty Detection

In the experiments of novelty detection, we aimed to detect new acoustic events by stacked AE, VAE, kNN, GMM, OCSVM, and iForest algorithms using Mel-spectrograms, and deep features extracted by TDNN, TDNN-LSTM, VGG, and ResNet networks. In Table 1, Table 2, Table 3 and Table 4, the average F1-scores of AND methods on each feature set extracted from the datasets were given for these AND scenarios for Domestic, ESC-10, US8K, and ESC-50. The best performances were observed on the deep features of VGG and F-TDNN through all the algorithms. Although several satisfactory performances were observed in stacked AE, kNN, and OCSVM algorithms, the GMM provided the best overall AND performance. Thus, in the experiment of the proposed ICL with AND approach, GMM was used.

**Figure 2:**
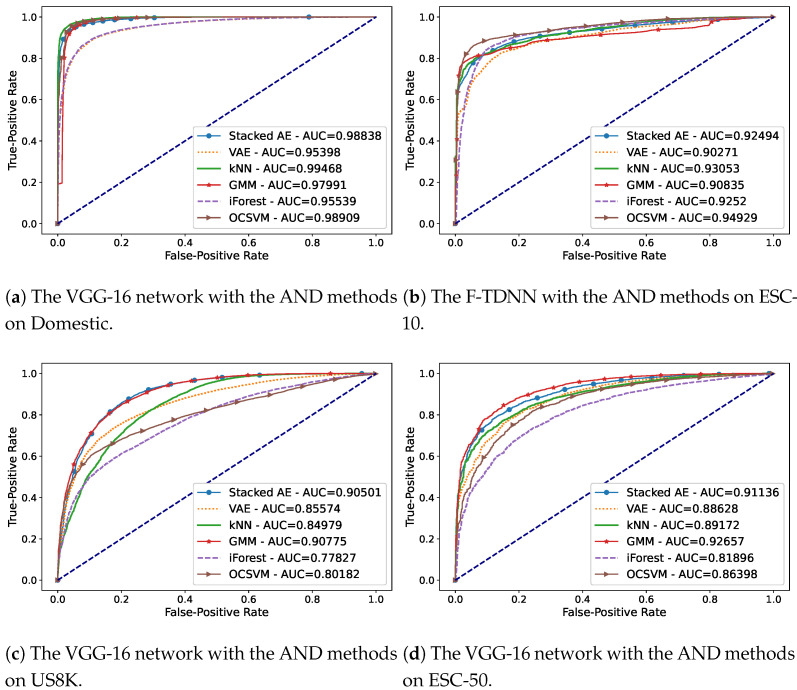
The AUC curves of the best performances obtained in the AND scenario in which only one event is known on the datasets: (**a**) Domestic, (**b**) ESC-10, (**c**) US8K, and (**d**) ESC-50.

Figure 2a–d, presents the best AUC scores obtained using an audio feature on each dataset when only one event was known. In this scenario, the features extracted by the VGG-16 network provided the best AND performances for Domestic (Figure 2a) and US8K (Figure 2c) datasets. In addition, using the features extracted from ESC-10 and ESC-50 shown in Figure 2b,d, the best AUC scores were observed combining F-TDNN and VGG with AND methods. The AND performances using the ResNet-34 features were mostly close to the best results, but in some scenarios withf a high amount of known classes, the AND methods had poor performances using the features.

**Figure 3:**
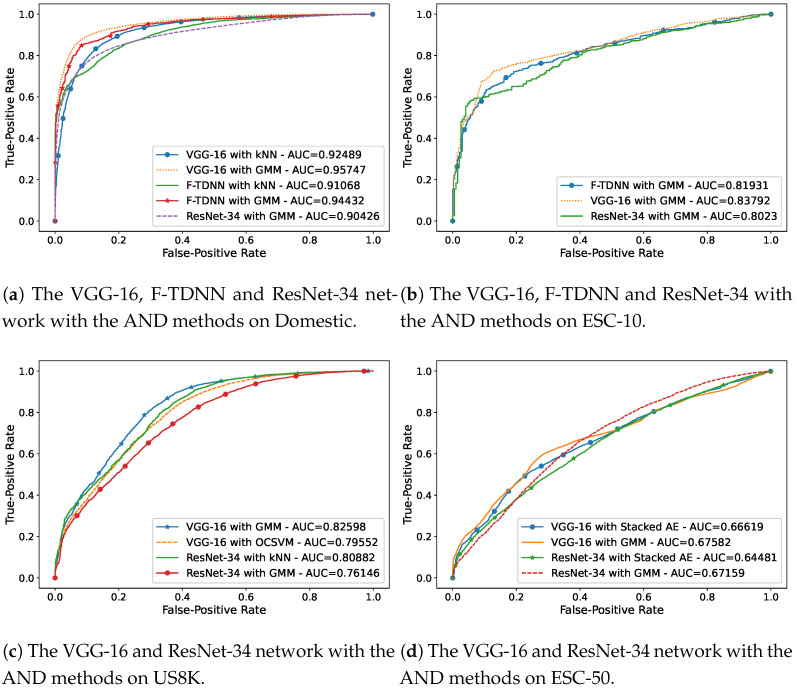
The AUC curves of the best performances of the AND methods in the most complicated AND scenario in which only one event is unknown on the datasets: (**a**) Domestic, (**b**) ESC-10, (**c**) US8K, and (**d**) ESC-50.

When most of the events were known, but only one event was unknown, VGG and F-TDNN provided the best novelty detection performances within most of the AND experiments. The AND performances of the features with the best AND methods are demonstrated in Figure 3a–d, which were obtained in the most complicated AND scenario; that is, only one event was unknown.

### 4.5. Results of the Incremental Class-Learning Experiments

In the ICL experiments, the performances of the LwF, iCaRL, and FearNet on the audio feature types were obtained without AND, while incrementally learning the rest of the acoustic event classes in a sequential way. The ICL performances were compared for accuracy changes observed using a test set including several samples of all the known events after each new event was learned.

**Figure 4:**
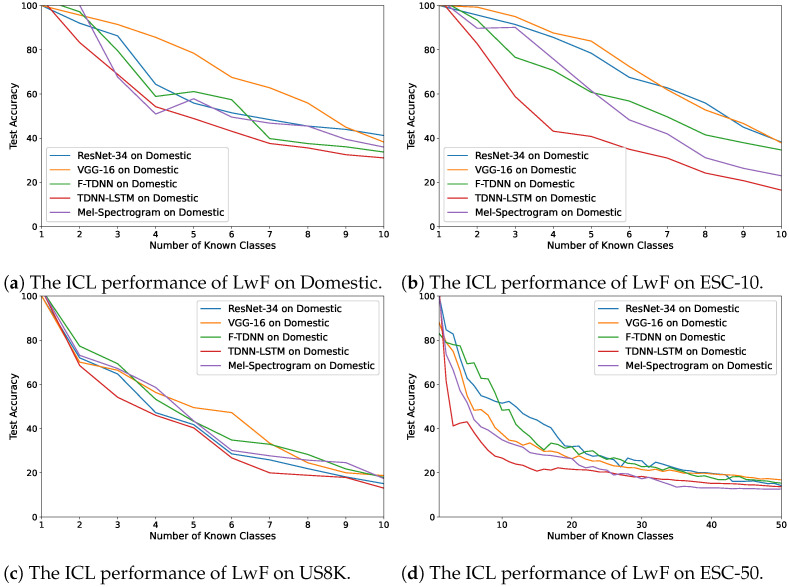
The average accuracy changes while incrementally learning new classes by LwF on the datasets: (**a**) Domestic, (**b**) ESC-10, (**c**) US8K, and (**d**) ESC-50.

Figure 4a–d demonstrates the changes in accuracy values while the LwF model was incrementally learning the classes. In our dataset, Domestic, ResNet features provided the best performance (Figure 4a), and VGG and ResNet obtained similar accuracy values after each class was learned (Figure 4b). Furthermore, in the most complex datasets (US8K and ESC-50) the deep features of VGG, ResNet and F-TDNN provided similar ICL performances as shown in Figure 4c,d. The accuracy values decreased less than 20% at the end. The worst ICL performance was obtained on each dataset using audio feature representations by TDNN–LSTM.

**Figure 5:**
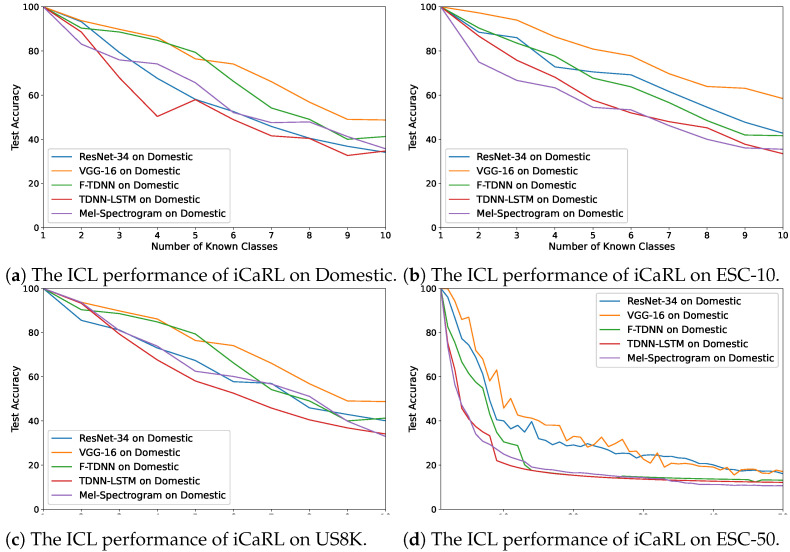
The average accuracy changes while incrementally learning new classes by iCaRL on the datasets: (**a**) Domestic, (**b**) ESC-10, (**c**) US8K, and (**d**) ESC-50.

**Figure 6:**
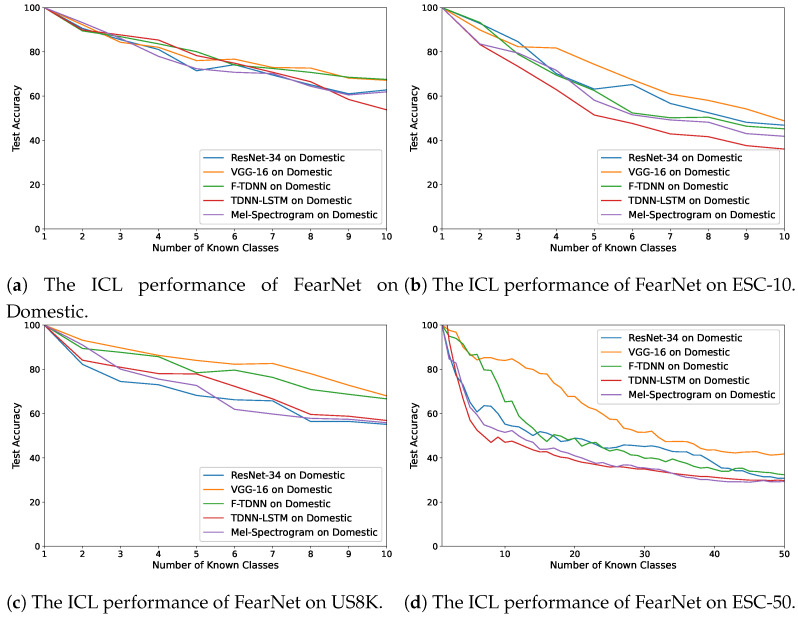
The average accuracy changes while incrementally learning new classes by FearNet on the datasets: (**a**) Domestic, (**b**) ESC-10, (**c**) US8K, and (**d**) ESC-50.

In Figure 5a–d, the accuracy changes obtained using iCaRL are demonstrated on these audio features. The best performance for each dataset was exhibited with the VGG features, and the performances with the VGG and ResNet features were similar when using the ESC-50, which comprised 50 distinct acoustic classes with several sounds. In addition, the worst performances of the algorithm with the Mel-spectrograms and the deep features of the TDNN–LSTM network were observed for each dataset. On the other hand, the FearNet algorithm achieved the highest performance using the deep features of VGG and F-TDNN (Figure 6a–d) compared with the other feature types. The best average accuracy value using the deep features of VGG and F-TDNN was by the FearNet algorithm (Table 5). For ICL, the deep representations provided better results than the Mel-spectrograms using all the ICL algorithms; therefore, the VGG and F-TDNN features were selected for the experiment of ICL with GMM.

The ICL performances of the FearNet algorithm on the datasets are demonstrated in Figure 6a–d. For most of the results, the FearNet provided the best overall performances on each dataset. However, the iCaRL algorithm also had suitable ICL performances, but it is observed that the previously learned acoustic events were forgotten while incremental learning using the LwF model. Therefore, in the last experiment, ICL with AND, the iCaRL, and FearNet algorithms were used to learn the new events detected by the GMM algorithm using the deep features extracted by the pre-trained VGG and TDNN (Section 4.6).

### 4.6. Results of Incremental Class-Learning with Novelty Detection

**Figure 7:**
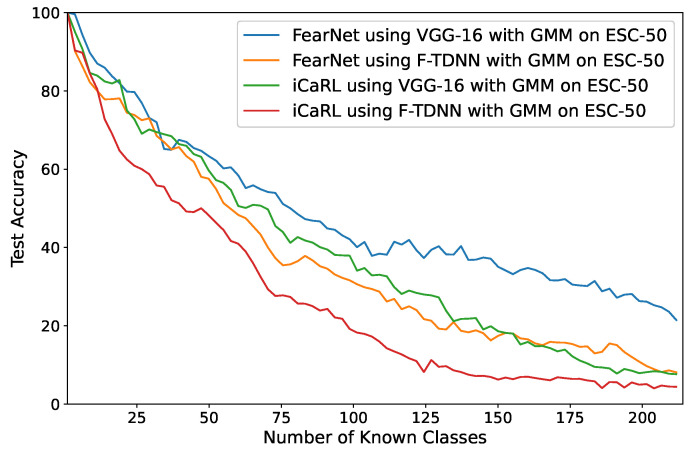
The performances of FearNet and iCaRL with GMM on ESC-50 in which the deep features, VGG, and F-TDNN of new classes detected by GMM are incrementally learned.

Using VGG and F-TDNN features, the FearNet and iCaRL algorithms were used for incremental learning on the new classes detected by GMM (Figure 1). In the AND step, multiple different audio samples of an event may have been detected as a new class. To evaluate the performances of ICL prediction, an audio sample of an event was labeled as a pseudo-label of the event, and the accuracy values were computed regarding the actual labels of the base events and predicted pseudo-labels. In Table 6, the average accuracy values of five different experiments using the proposed approach are listed in which each sample of an audio feature representation detected as a new class of acoustic events was incrementally learned. In Figure 7, the changes of accuracy values obtained after the detection of each new class are shown in which the best performance on the largest dataset (ESC-50) was obtained by the FearNet algorithm using VGG features similar to the ICL experiments. Furthermore, this experiment started with a randomly selected class, and the GMM detected 214 new acoustic events over time.

### 4.7. Discussion

The experimental evaluations demonstrated that a new acoustic class can be successfully detected and incrementally learned. The most suitable performances were presented by the algorithms using the VGG features. Unlike TDNN–LSTM, F-TDNN provided satisfying ICL performances on these datasets. For the ICL of the detected classes, the iCaRL method provided the best performance. Therefore, only audio information directly from the targets can be used to achieve ICL of detected novel acoustic classes.

In the ICL experiments, we showed that the performances of the ICL algorithms in the acoustic domain were comparable to the results in other domains such as computer vision, image processing or pattern recognition. Therefore, the ICL with AND approach can be used for various acoustic problems in which incremental learning of new tasks/classes may be required (e.g., bioacoustic [64], acoustic anomaly detection [55], or robot audition), and for multi-modal problems including sound such as audio-visual recognition tasks [65].

## 5. Conclusions

In this work, for the first time, the incremental class-learning (ICL) of acoustic events was developed and investigated on different types of audio features in benchmark audio datasets such as ESC-10, ESC-50, US8K, and our dataset, Domestic. Therefore, we proposed a novel ICL approach by integrating it with an acoustic novelty detection (AND) method for human-like lifelong learning. In this study, the AND was employed in a semi-supervised manner by retraining the AND model with features from the actual and augmented signals of the recently detected novel event class. In the experiments, the performances of the AND and ICL methods using five audio features, Mel-spectrograms, and deep features of the pre-trained F-TDNN, TDNN-LSTM, VGG-16, and ResNet-34 were evaluated to estimate the most appropriate feature types and algorithms for ICL with AND. In addition, this study is one of the few works on the extraction of embeddings of acoustic events by pre-trained TDNNs.

In the acoustic ICL tasks, new audio classes were detected by an AND algorithm, and then ICL wass achieved on the detected new classes, while the ICL was applied to the labeled novel classes in other ICL works.

Regarding future work, the number of acoustic event classes to be recognized will be increased to the order of hundreds and the performance of the proposed approach will be verified. Furthermore, due to the availability of a large amount of instances belonging to unknown classes in addition to previously learned classes, a semi-supervised method will be developed to incrementally learn the instances of unknown and known classes.

## Figures and Tables

**Table 1:** The Average F1/AUC Scores of AND on Domestic dataset.

Algorithm	Mel-Spectrogram	F-TDNN	TDNN-LSTM	ResNet-34	VGG-16
Stacked AE	89.7/92.1	91.7/94.0	86.1/88.3	90.1/92.9	92.8/95.0
VAE	83.5/86.2	84.1/85.2	80.7/81.7	87.3/90.3	88.9/91.1
kNN	86.0/88.2	91.1/94.7	81.5/83.8	88.8/90.4	94.7/**97.1**
GMM	92.7/94.4	**96.1**/**97.2**	86.5/86.7	92.3/94.2	**96.4**/**97.4**
OCSVM	86.3/90.6	86.1/91.2	80.1/87.5	85.1/91.7	91.4/94.9
iForest	78.4/81.6	77.4/84.1	74.9/76.8	80.7/86.1	83.1/88.1

**Table 2:** The Average F1/AUC Scores of AND on ESC-10 dataset.

Algorithm	Mel-Spectrogram	F-TDNN	TDNN-LSTM	ResNet-34	VGG-16
Stacked AE	83.1/86.8	76.4/83.1	70.4/74.3	81.5/86.1	81.4/88.7
VAE	76.0/83.2	76.1/83.4	66.4/67.8	83.8/86.3	81.3/85.2
kNN	81.1/88.2	84.8/86.9	70.7/73.8	83.8/87.2	88.4/**89.1**
GMM	80.5/87.9	85.1/**88.9**	77.0/77.7	83.0/86.7	**89.0**/**89.1**
OCSVM	80.5/85.8	78.1/81.1	76.2/74.1	83.1/85.0	86.2/88.3
iForest	67.2/71.4	62.4/65.2	59.4/57.8	63.3/66.3	71.3/73.0

**Table 3:** The Average F1/AUC Scores of AND on US8K dataset.

Algorithm	Mel-Spectrogram	F-TDNN	TDNN-LSTM	ResNet-34	VGG-16
Stacked AE	65.7/74.6	63.8/68.9	59.9/63.3	80.8/84.4	81.3/84.8
VAE	62.2/68.6	60.1/66.6	56.7/62.4	74.8/77.9	74.7/76.7
kNN	72.5/78.7	69.2/74.8	65.5/72.8	**83.5**/**87.1**	82.0/85.6
GMM	70.8/78.9	73.0/78.9	71.2/78.5	80.1/85.9	**85.1**/**87.7**
OCSVM	68.9/71.3	65.1/68.9	66.1/73.5	76.4/80.1	**84.1**/**87.3**
iForest	60.5/62.8	59.7/64.0	55.8/57.7	62.2/66.8	63.3/68.2

**Table 4:** The Average F1/AUC Scores of AND on ESC-50 dataset.

Algorithm	Mel-Spectrogram	F-TDNN	TDNN-LSTM	ResNet-34	VGG-16
Stacked AE	68.9/69.8	65.5/67.6	58.1/60.4	68.9/70.1	**71.6**/**72.7**
VAE	53.4/58.9	60.9/64.4	59.9/62.2	67.4/68.8	66.3/69.7
kNN	67.9/70.7	66.6/68.3	60.1/63.8	70.9/71.8	70.4/71.1
GMM	71.0/73.8	68.1/69.8	59.5/64.7	**71.2**/**73.4**	**71.9**/**73.4**
OCSVM	68.9/71.4	64.3/66.8	58.8/60.0	65.7/68.8	68.1/69.4
iForest	52.2/54.2	56.1/58.2	48.1/52.8	56.2/58.1	59.2/60.1

**Table 5:** The average accuracy values of the ICL algorithms using VGG/F-TDNN features in five experiments.

Algorithm	Domestic	ESC-10	US8K	ESC-50
LwF	69.4/64.8	64.2/60.0	57.1/54.4	24.1/20.9
iCaRL	78.5/77.6	68.1/68.3	62.1/59.6	28.1/21.1
FearNet	**80.7/81.4**	**74.3/71.0**	**63.8/59.5**	**30.8/24.7**

**Table 6:** The average accuracy values of the ICL algorithms with GMM using VGG/F-TDNN features and number of detected classes in three experiments.

	Accuracy Values on VGG/F-TDNN and Number of Detected Events			
Algorithm	Domestic	ESC-10	US8K	ESC-50
iCaRL	56.4/51.0/26	48.0/44.3/36	42.4/36.2/40	14.4/9.7/226
FearNet	**59.1**/**52.6**/26	**53.3**/**50.3**/36	**43.9**/**39.3**/40	**17.8**/**14.7**/226

## Data Availability

Our domestic dataset is available from the corresponding author on reasonable request. Also, the benchmark datasets, AudioSet, UrbanSound8K and ESC are available from: https://research.google.com/audioset/download.html (accessed on 12 February 2021), https://dataverse.harvard.edu/dataset.xhtml?persistentId=doi:10.7910/DVN/YDEPUT (accessed on 20 December 2020) and https://urbansounddataset.weebly.com/urbansound8k.html (accessed on 20 December 2020), respectively.
